# Validation of the geographic position of EPER-Spain industries

**DOI:** 10.1186/1476-072X-7-1

**Published:** 2008-01-11

**Authors:** Javier García-Pérez, Elena Boldo, Rebeca Ramis, Enrique Vidal, Nuria Aragonés, Beatriz Pérez-Gómez, Marina Pollán, Gonzalo López-Abente

**Affiliations:** 1 Cancer and Environmental Epidemiology Area, National Centre of Epidemiology, Carlos III Institute of Health, Madrid, Spain; 2 CIBER Epidemiología y Salud Pública (CIBERESP), Spain

## Abstract

**Background:**

The European Pollutant Emission Register in Spain (EPER-Spain) is a public inventory of pollutant industries created by decision of the European Union. The location of these industries is geocoded and the first published data correspond to 2001. Publication of these data will allow for quantification of the effect of proximity to one or more such plant on cancer and all-cause mortality observed in nearby towns. However, as errors have been detected in the geocoding of many of the pollutant foci shown in the EPER, it was decided that a validation study should be conducted into the accuracy of these co-ordinates. EPER-Spain geographic co-ordinates were drawn from the European Environment Agency (EEA) server and the Spanish Ministry of the Environment (MOE). The Farm Plot Geographic Information System (Sistema de Información Geográfica de Parcelas Agrícolas) (SIGPAC) enables orthophotos (digitalized aerial images) of any territorial point across Spain to be obtained. Through a search of co-ordinates in the SIGPAC, all the industrial foci (except farms) were located. The quality criteria used to ascertain possible errors in industrial location were high, medium and low quality, where industries were situated at a distance of less than 500 metres, more than 500 metres but less than 1 kilometre, and more than 1 kilometre from their real locations, respectively.

**Results:**

Insofar as initial registry quality was concerned, 84% of industrial complexes were inaccurately positioned (low quality) according to EEA data versus 60% for Spanish MOE data. The distribution of the distances between the original and corrected co-ordinates for each of the industries on the registry revealed that the median error was 2.55 kilometres for Spain overall (according to EEA data). The Autonomous Regions that displayed most errors in industrial geocoding were Murcia, Canary Islands, Andalusia and Madrid. Correct co-ordinates were successfully allocated to 100% of EPER-Spain industries.

**Conclusion:**

Knowing the exact location of pollutant foci is vital to obtain reliable and valid conclusions in any study where distance to the focus is a decisive factor, as in the case of the consequences of industrial pollution on the health of neighbouring populations.

## Background

The European Pollutant Emission Register in Spain (EPER-Spain) [1] is a public inventory of Spanish companies coming within the scope of application of the Integrated Pollution Prevention and Control (IPPC) Act 16/2002 [2]. It includes all industrial and livestock-sector installations that have acknowledged exceeding the reporting thresholds for one or more of the pollutants listed in European Union Decision 2000/479/CE. The first data published, corresponding to 2001, included 1,437 companies. An initial analysis of this information, which includes a preliminary quantification of reported pollutants released plus a comparison between Spanish and European data, has recently been published [3].

One of the novel features introduced by this registry is that, in addition to furnishing data on pollutant emissions, it includes the geographic location of each facility, providing both the postal address and its geographic co-ordinates.

This information can be extremely useful when studying the possible effect of these industries on the health of neighbouring populations (as shown by other studies [4-12]), since it serves to improve analyses that link geographic morbidity and mortality patterns to the presence of pollutant foci. Yet, its utility depends, in part, on the quality of the geocoding of such industries, i.e., on whether the geographical location reflected in the registry is in fact correct.

EPER-Spain industrial co-ordinates can be obtained from two sources, namely, the European Environment Agency (EEA) and the Spanish Ministry of the Environment (MOE). This information was compared against the Farm Plot Geographic Information System (Sistema de Información Geográfica de Parcelas Agrícolas) (SIGPAC).

Geocoding methods and data-validation processes in epidemiological studies have attracted a lot of attention in recent years. The accuracy of these data could be a critical point if increases in risk are identified in populations living close to pollutant facilities.

A preliminary analysis of the EPER-Spain highlighted the existence of errors in the location of many facilities. To assess the quality of this information, we decided to validate the geographic co-ordinates of all industrial-sector companies included in the EPER-Spain (639 industrial facilities). For the purpose, the information contained in the SIGPAC was used and supplemented with other locally available resources to geocode each industry anew, with the aim of identifying incorrectly plotted facilities and studying the degree of error between the real co-ordinates and those included in the official registries.

## Results

Information was successfully obtained as to the exact location of EPER-Spain's 639 industrial facilities.

**Figure 1:**
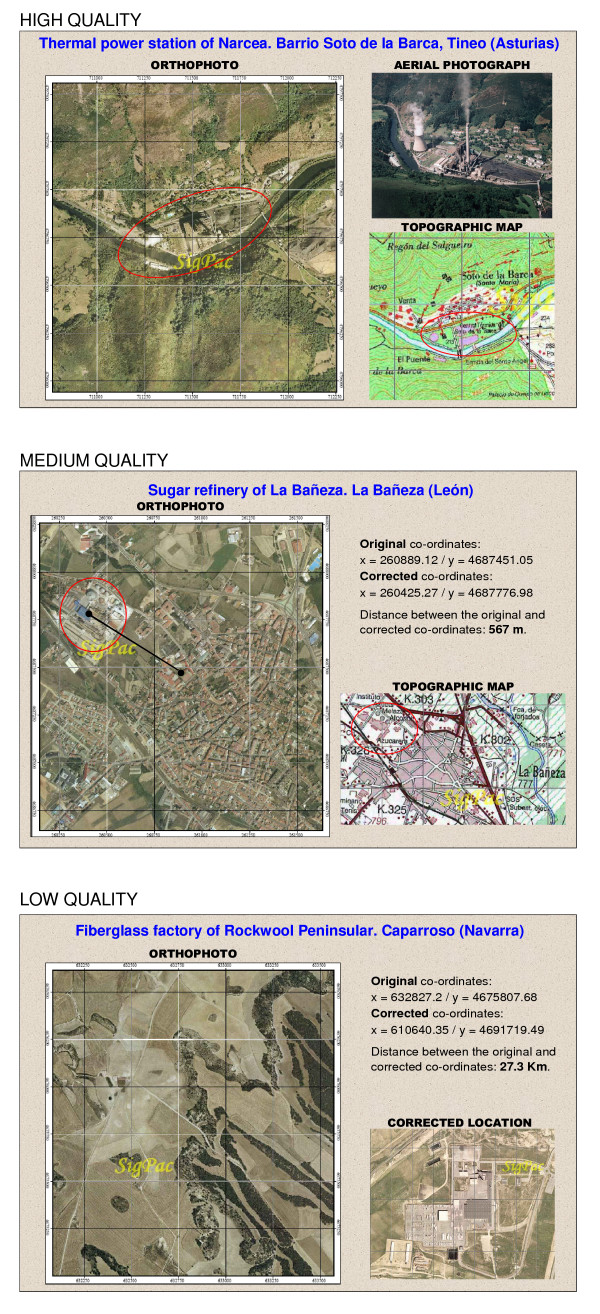
Examples of industries broken down by different quality criteria to identify their geographical location.

Figure 1 depicts three examples of industries whose situation was classified according to the quality criteria outlined above. In the first example, i.e., high quality, the facility is shown in the centre of the orthophoto. The topographic map furnished by the SIGPAC (in which the name of the industrial complex appears), as well as the aerial photograph confirm that this is the industry sought. The second example shows an industry with medium-quality geocoding. The last example is an instance of low quality, in which the orthophoto obtained from the original co-ordinates shows no industrial plant.

**Figure 2:**
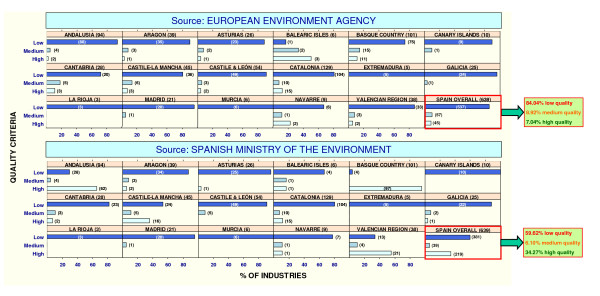
Initial quality of EPER-Spain industry geocoding.

Figure 2 shows the quality of the official geocoding of industries by the two data sources used (the EEA and the Spanish MOE). The graphs depict the percentage distribution of industries according to the defined quality criteria and the absolute number of industries in each category for each Autonomous Region and for Spain as a whole.

Only 7% of industries were accurately located (high quality), using information from the EEA, a percentage that rose to 34% when we used data from the 2006 update furnished by the Spanish MOE. Overall, data sourced from the Spanish web page were of better quality than the European data. Analysis by Autonomous Region highlighted the fact that this difference was due to the effort made by certain regional authorities, the bodies required to report this information: specifically, in the Basque Country the number of accurately situated industries rose from 11% to 96%. Further Autonomous Regions with relevant improvements in their data were Andalusia, the Valencian Region and Castile-La Mancha. In contrast, other regions displayed very poor quality information in both sources, with 100% of their industries inaccurately positioned (e.g., Murcia, La Rioja and Extremadura).

Table 1 summarises the distribution of distances (in kilometres) between the original location of the industries and their correct location, according to the co-ordinates supplied by the two servers, for each Autonomous Region and for Spain overall. The median distance between the corrected location and the data furnished by the EEA was 2.55 kilometres for the whole of Spain, though there was wide variability among the different Autonomous Regions. While the Balearic Islands registered the smallest difference between the two co-ordinates, with a median distance of 0.57 kilometres, this measure was in excess of 6 kilometres in Murcia and the Canary Islands (6.48 and 6.17 kilometres respectively). Special mention should be made of errors of note, such as the case of the industrial plant owned by *Desgasificación y limpieza de tanques S.A*., which was shown to be situated at a distance of 780.90 kilometres from its real location, possibly due to an error in data entry.

**Table 1:** Percentiles of distribution of the distances between original and corrected EPER-Spain industry co-ordinates, shown by Autonomous Region and for Spain overall (in km).

		Source: European Environment Agency						Source: Spanish Ministry of the Environment					
	n	P 10	P 25	P 50 (median)	P 75	P 90	Maximum	P 10	P 25	P 50 (median)	P 75	P 90	Maximum
**SPAIN**	**639**	**0.72**	**1.45**	**2.55**	**4.71**	**7.51**	**780.90**	**0.00**	**0.00**	**1.63**	**3.53**	**6.79**	**269.80**
ANDALUSIA	94	1.48	2.63	4.34	6.69	9.28	780.90	0.00	0.00	0.00	2.01	6.05	140.12
ARAGON	39	1.09	1.70	3.43	5.52	7.00	9.43	0.87	1.86	3.32	5.42	7.38	21.73
ASTURIAS	26	0.89	1.76	3.91	5.77	10.43	12.89	1.36	2.99	4.11	5.72	10.59	14.73
BALEARIC ISLES	6	0.24	0.39	0.57	0.89	3.33	5.71	0.33	1.20	4.25	6.42	6.98	7.30
BASQUE COUNTRY	101	0.48	1.00	1.63	2.59	3.86	24.01	0.00	0.00	0.00	0.00	0.00	84.62
CANARY ISLANDS	10	1.93	3.06	6.17	11.24	38.49	271.98	2.38	2.57	3.18	7.79	45.41	269.80
CANTABRIA	28	0.45	0.95	1.73	2.35	3.25	5.67	0.71	1.29	1.99	2.72	3.43	5.67
CASTILE-LA MANCHA	45	0.69	1.14	2.72	3.72	6.40	653.08	0.00	0.00	1.10	2.72	3.63	9.99
CASTILE & LEÓN	54	1.15	2.20	3.31	4.85	6.90	128.49	1.12	2.05	3.36	4.42	5.59	12.70
CATALONIA	129	0.35	1.14	2.17	3.63	6.96	27.38	0.35	1.14	2.17	3.63	6.96	27.38
EXTREMADURA	5	1.13	1.16	1.63	4.34	8.67	11.55	1.13	1.15	1.63	4.35	8.67	11.55
GALICIA	25	1.54	1.66	2.79	5.57	8.31	9.88	0.87	1.61	2.55	3.88	7.33	10.09
LA RIOJA	3	1.47	1.81	2.38	2.64	2.79	2.89	1.29	1.63	2.21	2.51	2.69	2.81
MADRID	21	1.65	2.81	4.76	6.22	16.65	127.58	1.73	2.15	3.71	6.22	11.75	92.96
MURCIA	6	3.44	4.11	6.48	7.42	10.97	14.46	3.52	4.03	5.86	7.18	11.27	15.06
NAVARRE	9	0.33	0.83	2.29	3.66	4.92	4.98	0.40	1.32	2.94	7.55	11.72	27.30
VALENCIAN REGION	38	0.91	1.59	2.52	3.53	5.60	7.49	0.00	0.00	0.00	1.41	3.09	22.66

In the case of data furnished by the Spanish MOE, the median error decreased to 1.63 kilometres for Spain as a whole, though there continued to be wide geographical variability in the quality of the information. The Autonomous Regions with worst quality data were Murcia (median error of 5.86 kilometres), Balearic Isles (median error of 4.25 kilometres) and Asturias (median error of 4.11 kilometres). In contrast, there were Autonomous Regions, such as the Basque Country, Andalusia and Valencian Region, which registered a median error of 0 kilometres, thereby reinforcing the fact that quality of the geographic co-ordinates of industrial facilities shown on the Spanish MOE server is higher than that of the EEA server, though both sources were far from achieving optimal geocoding of EPER-Spain industries.

By way of illustration, Table 2 lists the statistics of the distribution of distances (in kilometres) by EPER industrial activity and industrial activity group. The 'Waste management' group of industries registered the greatest errors in both sources studied.

**Table 2:** Percentiles of distribution of the distances between the original and corrected EPER-Spain industry co-ordinates, shown by industrial activity and industrial activity group (in kilometres).

		Source: European Environment Agency						Source: Spanish Ministry of the Environment					
	n	P10	P 25	P 50	P75	P 90	Maximum	P10	P 25	P 50	P75	P 90	Maximum
**TOTAL GROUP 1: ENERGY INDUSTRIES**	**69**	**0.71**	**1.69**	**3.74**	**5.71**	**7.93**	**271.98**	**0.00**	**0.17**	**2.06**	**4.16**	**6.83**	**269.80**
Combustion installations (> 300 MW)	27	1.61	4.54	5.72	7.88	12.55	27.00	0.28	2.48	5.27	5.71	10.09	27.00
Combustion installations (> 50 and < 300 MW)	18	1.36	2.07	3.48	5.17	9.20	271.98	0.00	1.92	2.63	4.81	11.02	269.80
Combustion in gas turbines	10	1.45	1.89	3.99	4.54	6.04	8.73	0.00	0.15	2.17	2.87	3.70	7.30
Combustion in stationary engines	4	0.29	0.53	0.91	1.29	1.53	1.70	0.10	0.26	0.51	0.93	1.39	1.69
Mineral oil and gas refineries	10	1.59	2.11	4.19	6.98	7.56	8.12	0.00	0.00	0.48	2.72	7.54	8.12
**TOTAL GROUP 2: PRODUCTION AND PROCESSING OF METALS**	**117**	**0.50**	**1.05**	**2.10**	**3.64**	**5.81**	**24.01**	**0.00**	**0.00**	**0.67**	**2.64**	**4.61**	**84.62**

Production of primary and secondary metals or sintering installations	17	1.02	1.69	2.87	4.93	6.89	12.89	0.00	1.18	3.01	4.31	5.01	14.73
Characteristic processes in the manufacture of metals and metal product	50	0.36	0.90	1.62	3.76	6.37	24.01	0.00	0.00	0.00	1.40	3.50	22.61
Surface treatment of metals and plastics	50	0.77	1.21	2.29	3.13	5.55	13.38	0.00	0.00	1.05	2.62	5.32	84.62
**TOTAL GROUP 3: MINERAL INDUSTRIES**	**141**	**0.79**	**1.39**	**2.72**	**3.86**	**6.28**	**653.08**	**0.00**	**0.00**	**1.44**	**2.95**	**5.02**	**139.22**

Manufacture of plaster, asphalt, concrete, cement, glass, fibres, bricks, tiles or ceramic products	141	0.79	1.39	2.72	3.86	6.28	653.08	0.00	0.00	1.44	2.95	5.02	139.22
**TOTAL GROUP 4: CHEMICAL INDUSTRY**	**115**	**0.77**	**1.58**	**2.70**	**4.73**	**7.21**	**14.46**	**0.00**	**0.00**	**2.11**	**3.55**	**6.88**	**15.06**

Manufacture of basic organic chemicals	61	0.77	1.56	2.45	4.69	7.00	14.46	0.00	0.90	2.21	4.08	7.14	15.06
Manufacture of basic inorganic chemical products or fertilisers	34	1.56	2.47	3.68	5.65	8.00	9.43	0.00	0.00	1.61	3.53	6.12	10.28
Manufacture of biocides and explosives	5	1.27	1.45	1.58	1.85	3.27	4.21	0.00	0.00	1.45	2.48	3.52	4.21
Manufacture of pharmaceutical products	15	0.29	0.98	2.08	3.55	3.72	11.81	0.00	0.47	2.15	3.41	3.59	11.68
**TOTAL GROUP 5: WASTE MANAGEMENT**	**60**	**1.39**	**2.49**	**4.56**	**7.76**	**11.73**	**780.90**	**0.00**	**0.94**	**3.16**	**6.90**	**11.84**	**27.38**

Installations for incineration of hazardous or municipal waste	8	1.63	2.28	2.98	5.40	10.56	18.82	2.27	2.51	4.11	6.74	8.44	11.75
Installations for physico-chemical and biological treatment of waste	6	1.44	3.69	10.09	16.00	22.01	27.38	1.44	3.70	10.03	14.02	20.73	27.38
Installations for regeneration/recovery of waste materials	7	1.52	1.62	2.53	5.47	316.79	780.90	0.00	0.00	1.01	1.42	1.71	1.88
Installations for the disposal of non-hazardous waste and landfills	39	1.70	3.35	5.25	7.81	9.27	13.71	0.00	0.48	3.36	6.91	8.98	12.94
**TOTAL GROUP 6: OTHER ACTIVITIES**	**137**	**0.67**	**1.39**	**2.12**	**4.53**	**6.58**	**128.49**	**0.00**	**0.00**	**1.48**	**3.55**	**6.78**	**140.12**

Installations for the manufacture of paper, pulp and paper products	40	0.69	1.14	1.73	4.76	8.31	128.49	0.00	0.00	1.18	3.58	9.99	140.12
Plants for the pre-treatment of fibres or textiles	15	0.78	1.61	2.23	2.63	8.64	26.12	0.00	0.00	0.00	1.68	2.66	9.93
Slaughterhouses, installations for the production of milk and other animal or vegetable raw materials	51	0.55	1.21	1.98	4.63	6.33	16.12	0.00	0.58	1.68	3.95	5.81	15.03
Application of paint in installations for surface treatment or products using organic solvents	22	1.51	1.93	2.47	3.39	5.41	127.58	0.00	0.00	2.14	4.93	7.53	92.96
Other activities (installations for disposal or recycling of animal waste, production of carbon or graphite, printing industries, and degreasing for surface treatment with solvents)	9	1.43	1.65	2.48	3.39	4.62	4.98	0.78	1.34	2.21	3.48	4.89	5.21

## Discussion

This study reports the results of assessing the data quality of the geographic co-ordinates for EPER-Spain industrial complexes, obtainable from the official European and Spanish web pages. Our results highlight both the poor overall quality and the wide variability in quality in Spain. Similarly, the data reflect the different attitudes adopted by the regional authorities in this regard. Although there has been an improvement in quality between the initial and 2006-updated data, these changes are restricted to certain specific regions, and the Basque Country, Andalusia, Valencian Region and Castile-La Mancha in particular.

The errors detected in the geographic co-ordinates of industrial installations are attributable to one or more of several reasons, namely:

1) Different industries situated in a single town being allocated the same geographic co-ordinates, e.g., as in the case of municipal centroid reporting.

2) Industries having the same name as the parent company being sited at the same point, e.g., as in the case of companies having a number of plants situated at different sites.

3) Spain is divided into the following 4 Universal Transverse Mercator (UTM) areas (zones): 28 (Canary Islands); 29 (Galicia and Western Asturias, Castile & León, Extremadura and Andalusia); 30 (central Spain); and 31 (Catalonia, Balearic Isles, eastern Aragon and the Valencian Region). There is an equivalence among the zones, and it is likely that industries may have committed errors in calculations leading to their complexes being situated in zone 30, which has been used as reference for the reporting of co-ordinates nationwide. Other reason for this potential problem could be the use of a wrong projection or not communicating the projection that has been used by the facilities.

4) Plants in extensive industrial areas being located at the same point (centre of the zone), where poor geocoding of this type can lead to errors amounting to kilometres, e.g., as in the case of industrial areas extending over several kilometres devoted to petroleum processing, where there are mineral oil and gas refineries, chemical industries and co-generation plants.

5) Industries belonging to the same industrial estate being located at the same geographical point. Here, the errors, albeit not quite as large as in the above case, can nevertheless amount to several hundreds of metres.

6) Geocoding of industries situated hundreds of kilometres away, in provinces or Autonomous Regions that do not correspond to the correct location: this problem would appear to stem from data-entry errors.

Although we have no evidence of the quality of the geographical data supplied by other EPER member countries, the possibility cannot be rule out that these may display similar problems to those of the EPER-Spain, given that quality assurance is in all cases the responsibility of the Member States and the industries subject to reporting. The European Commission and the EEA only conduct a limited verification of certain quality aspects linked to the completeness and coherence of reported data. In our case, both the co-ordinates and the remaining mandatory data (pollutant quantities released, address of complexes, number of workers, hours of production and type of industrial activity) are reported by the industries to the Regional Environmental Authorities, and it would be advisable if such data were submitted to quality control before being sent to the EEA.

One of the aspects that may have influenced the initial quality of EPER-Spain geographic co-ordinates stems from the indications included in the EPER Directive as regards the geocoding of facilities. The Guidance Document for EPER implementation proposes the address (street name and number, and postal code) and the co-ordinates as mandatory fields for the geographic location of the industrial complex. In addition, it proposes that, "The co-ordinates should be expressed in longitude and latitude co-ordinates (to be read from a topographic map in degrees and minutes, giving a precision of the order of one kilometre and referring to the geographical centre of the site of the facility)" [13]. This marked precision of one kilometre could be insufficient for positioning companies correctly.

The EPER is soon to be replaced by the European Pollutant Release and Transfer Register (E-PRTR), which will include more comprehensive information on industrial pollution from 91 substances and 65 industrial activities, as well as information on waste management by industrial installations. It will also compile pollutionreports from a range of sources, such as road and air transport, shipping and agriculture. It should be noted that the Guidance Document for the implementation of the European PRTR [14] proposes that, "The co-ordinates of the location should be expressed in longitude and latitude co-ordinates giving a precision of the order of at least ± 500 metres and referring to the geographical centre of the site of the facility". This will amount to an improvement in precision with respect to the EPER and will very probably have a positive impact on the geocoding of complexes in forthcoming reports. Furthermore, this precision of 500 metres coincides with the high-quality criterion used in our study to obtain orthophotos of industrial complexes.

One of the key factors in this study was the use of the SIGPAC for locating pollutant foci. This is a rigorous, reliable and updated Geographic Information System (GIS) that provides numerous details about the geography of Spain. Attention should be drawn to the fact that the data reported by industries correspond to 2001 and that the dates of the flights which produced the SIGPAC orthophotos correspond to 2001–2002.

One limitation of the SIGPAC is the information furnished by its topographic maps. Although the names of industries, industrial estates, roads, streets and buildings appear, this information is not available for all industries or for all towns. Almost all the major complexes (thermal power stations, mineral oil and gas refineries, chemical industries, cement industries, metallurgical industries and automobile plants) are identified in the topographic maps, as are a great many middle-sized industries (paper mills, incinerators and chemical facilities). Yet, the names of most of the small industries (slaughterhouses, tile works, textile factories, small-sized metal production and processing plants or landfills) do not appear. To compensate for this shortcoming and to check the accuracy of the SIGPAC vis-à-vis the remaining complexes, we resorted to other means for help (described under the Methods section).

The methodology envisaged in this study could be applied to the validation of EPER-Spain companies engaged in the livestock sector (farms, organic manure management), though this step would be fairly laborious, due to the fact that these are small facilities whose names are not shown on the SIGPAC topographic maps in the majority of cases.

Methods or processes for geocoding co-ordinates and validating data in epidemiological studies have recently attracted a considerable amount of attention. While some studies have examined different methods of geocoding address co-ordinates [15-23], others have assessed the effect of positional error when automatic geocoding methods are used [20,24-26] or different errors in the geocoding process [27,28].

Studies on this topic have recently been published, evaluating the importance of correct geocoding in environmental studies [22,24]. In spatial-epidemiology studies, allocation of geographic co-ordinates may lead to a given area being subsequently classified in accordance with other variables for which there is information at a geographic level (e.g., allocation of a specific socio-economic level in accordance with the census section to which it belongs). Accumulation of classification errors renders assessment of the precision or coherence of the final result difficult [22,24,29].

**Figure 3:**
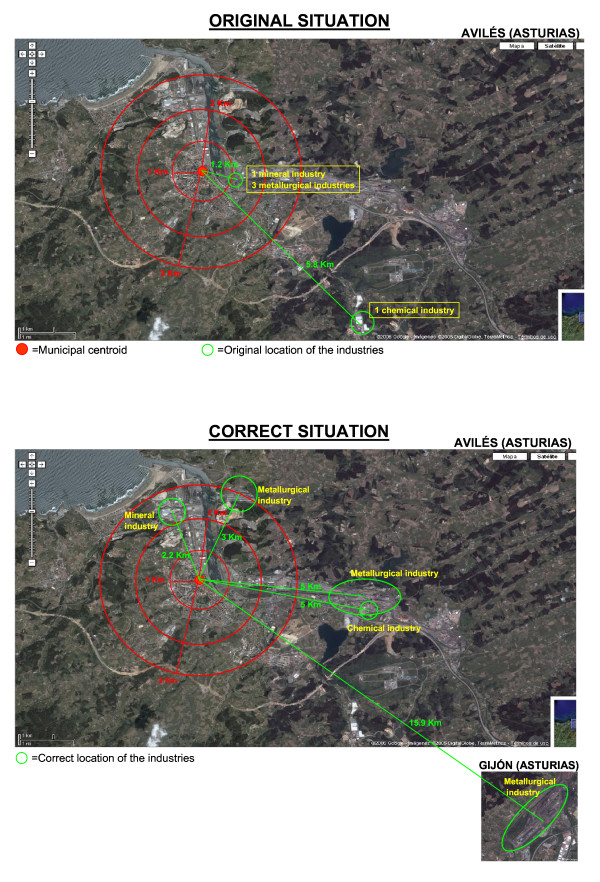
Example of poor geocoding applied to the town of Avilés (Asturias).

Figure 3 is an example of the way in which inaccurate geocoding can negatively affect pollutant foci in a study on the health consequences of industrially generated pollution for populations living in the environs of such facilities. The original situation corresponds to the data furnished by the EEA for the town of Avilés, where 5 industries appear, whilst the second image corresponds to the correct location. If one wished to study the effect produced by pollutant foci in a radius of 2 kilometres around the centroid of Avilés, no industry would be included in such a study, whereas in the original (i.e., the incorrect) situation, 4 industries would be included. Taking a radius of 3 kilometres around the municipal centroid, 2 industries would be included in the study, whereas in the original (i.e., the incorrect) situation, 4 pollutant foci would be included. In this case, exposure would be overestimated if one were to choose the original EPER-Spain file co-ordinates, since industries would be included that were not really as close to the centroid as they appear to be. Lastly, it should be pointed out that one of the industries which was originally situated in Avilés actually corresponds to Gijón, so that if the original co-ordinates were chosen, one would be underestimating exposure to pollutant foci in the latter city.

Finally, we want to highlight some interest aspects derived from this validation study:

1) Data entry problems, like the errors in the location or geocoding of many facilities shown in the EPER-Spain may cause large errors in subsequent studies that use these data. The expenses of double entry protocols or validation studies are justified by the observed data entry problems and subsequent errors.

2) The local efforts of regional authorities and high standards of accuracy can lead to improve the quality of the geocoding of the industries, as observed in some Autonomous Regions in Spain.

3) The common use of municipal centroids instead of the exact co-ordinates by many of the facilities is inadequate.

## Conclusion

Our results highlight the errors in the information furnished by the EPER, on its official web pages, both European and Spanish, though there has been an improvement in quality between the initial data and those updated in 2006. There is great variability within Spain, with the Basque Country, Andalusia, Valencian Region and Castile-La Mancha being the Autonomous Regions that furnish the best-quality data. There are methods, the SIGPAC in particular, which allow for all industries to be accurately located.

Knowing the exact location of pollutant foci is vital to obtain reliable and valid conclusions in any study where distance to the focus is a decisive factor, as in case of the consequences of industrial pollution on the health of neighbouring populations.

## Methods

EPER-Spain industrial co-ordinates are obtainable from two sources. In February 2004, the EEA, acting through the EPER registry in Europe [30] published the co-ordinates of EPER-Spain industries, using 2001 data based on information furnished by the respective Environmental Authorities of Spain's Autonomous Regions. Subsequently, as a consequence of the application of Spanish legislation [2], the Autonomous Regions sent updated information on the industries to the Spanish MOE, which in turn disseminated this via the EPER's Spanish-based web page in 2006 [1].

EPER-Europe and EPER-Spain furnish geographic WGS84-projection co-ordinates (longitude/latitude). These co-ordinates were converted into UTM Zone 30 (ED50) co-ordinates (X, Y) and incorporated into a GIS.

Currently, there are different tools that enable any point of Spanish territory to be accurately located. In a first phase, the quality of registry data was evaluated using the SIGPAC, a GIS belonging to the Spanish Ministry of Agriculture, Fisheries & Food. This system is designed to monitor Common Agricultural Policy (CAP) grants and includes orthophotos of the entire surface of Spanish territory, along with topographic maps showing the names of industries, industrial estates, roads, buildings and streets [31]. An orthophoto is a photographic depiction of an area of the Earth's terrestrial surface, on which all the elements are shown error- and distortion-free on the same scale, with the same validity as a cartographic plan. In other words, it can be regarded as a photograph that displays the images of objects in their true orthographic position, and is geometrically equivalent to a plan [32]. Although an orthophoto is an image, its geometrical precision and radiometrical accuracy are of crucial importance [33].

The SIGPAC enables any point of Spanish geography to be visualised, whether by searching directly (by region, province, town, industrial estate or plot) or by co-ordinates (by UTM, X and Y co-ordinates, zone and radius of visualisation of the point sought).

The initial UTM co-ordinates of each of the industries were fed into the SIGPAC and orthophotos were obtained with various radiuses of visualisation. The original location was classified using the following quality criteria:

1) high quality, where industrial facilities were really located at a distance of less than 500 metres from the centre of the orthophoto (which corresponds with the original co-ordinates);

2) medium quality, where industrial facilities were shown to be situated more than 500 metres but less than 1 kilometre from the centre of the orthophoto; and,

3) low quality, where industrial facilities were shown to be situated at a distance of more than 1 kilometre from the centre of the orthophoto.

Based on the orthophotos, and using the information from the topographic maps, we plotted the exact location of those industries whose co-ordinates were not correct and corroborated the location of those whose initial co-ordinates were correct.

Facilities whose SIGPAC situation was in doubt were located using other means, such as the GoogleMaps server [34] (which allows for a search of addresses and companies, and offers high-quality aerial photographs), yellow pages web page [35] (which allows for a search of addresses and companies), Internet aerial photographs, and the web pages of the industries themselves (e.g., web page of Spanish cement industries [36]) and various local and regional institutions.

Industry percentages, broken down by the respective quality criteria, are shown for each Autonomous Region and for Spain as a whole. For each of the industries studied, the distance between the corrected and original European and Spanish registry co-ordinates was also calculated and a descriptive analysis of this information was performed for each Autonomous Region, for Spain overall, and for the different activities and industrial groups.

## Competing interests

The author(s) declare that they have no competing interests.

## Authors' contributions

JGP and GLA conceived the idea and JGP wrote the manuscript. EB contributed to manuscript writing. EB, RR, EV, NA, BPG, MP and GLA revised the manuscript for important intellectual content. All authors contributed to the final version of the manuscript.
